# Study on the mechanism of methane “solid–liquid–gas” conversion controlled by the evolution of coal micro- and nanopore structure

**DOI:** 10.1038/s41598-024-62193-x

**Published:** 2024-05-20

**Authors:** Hao Sui, Xijian Li, Junjie Cai, Sen Deng, Enyu Xu, Feng Xue, Honggao Xie

**Affiliations:** 1 College of Resource and Environmental Engineering, Guizhou University, Guiyang, 550025 China; 2 Mining College, Guizhou University, Guiyang, 550025 China; 3 Guizhou Engineering Center for Safe Mining Technology, Guiyang, 550025 China

**Keywords:** Tectonic coals, Pore structure, Adsorption capacity, Molecular simulation, Energy storage, Geology, Mineralogy

## Abstract

Currently, the utilization of coalbed methane resources in the Guizhou region faces challenges such as complex reservoir structure, high gas content, and microporous development. Based on these, the pore structure and adsorption capacity of Guizhou tectonic deformed coals (TDCs) were evaluated using a suite of integrated diagnostic techniques including low-temperature nitrogen adsorption (LT-N_2_A), mercury intrusion porosimetry (MIP), methane isothermal adsorption. Through the above methods, the pore structure and adsorption characteristics of the samples were characterized; The samples were divided into the range of joint pores by combining the results of MIP and LT-N_2_A; Using the molecular simulation software, the 2 nm, 4 nm, 10 nm pores affecting the methane endowment state were investigated respectively, and from the perspective of the heat of adsorption and energy, the concept of the three-phase transition of methane was proposed, and explore the change of the pore spacing affecting the endowment state of methane from the solid state pore to the gas state pore. The results provide new ideas for the in-depth study of gas storage in tectonic coal reservoirs in Guizhou Province.

## Introduction

Coalbed methane (CBM) resources have great potential and broad prospects for development and utilization in Guizhou. However, most of the coal seams have poor permeability and low gas saturation, so the gas management effect of conventional extraction measures is poor, and low extraction efficiency is the bottleneck affecting safe mining. In recent years, with the development of reservoir reforming technology, the relevant process has effectively improved the permeability of coal seams, but the problem of declining extraction efficiency and extraction volume of reservoirs in the late stage of development still can't be improved^1^. Studies have shown that the micro-nano pores of coal in the Guizhou region are developed, and the gas adsorption capacity is strong, it is difficult to desorb effectively^2–4^. This leads to the fact that the existing technical means in Guizhou region cannot effectively exploit CBM on a large scale. The key reason is that the mechanism of methane phase transition in coal reservoirs with micro- and nanopore development in Guizhou is not well understood, so the effectiveness of the existing methane desorption methods is insufficient.

**Figure 1:**
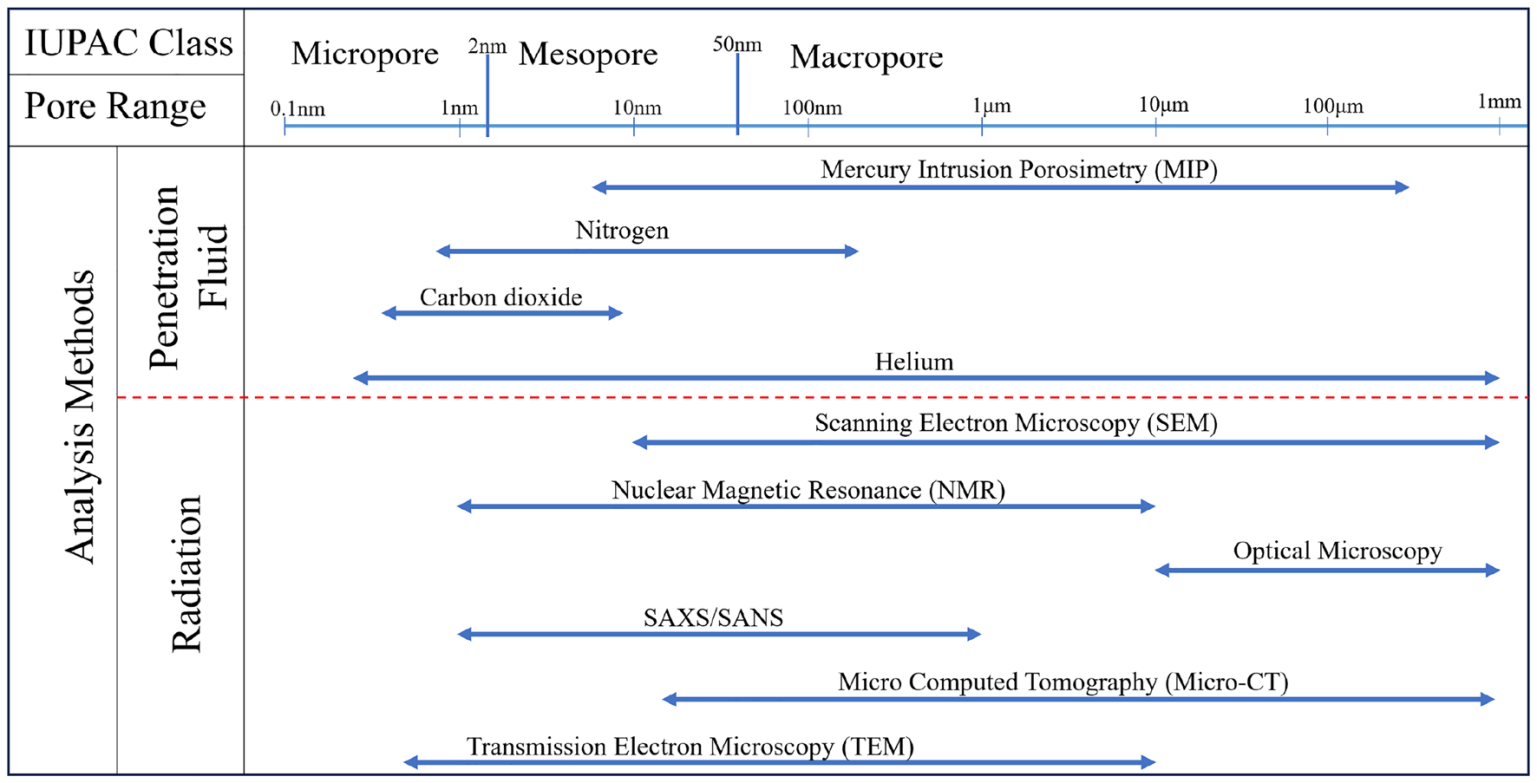
Analysis methods and the ranges of application of pore structure^12,13^.

Methane adsorption and desorption behaviors take place in coal pores, so the pore structure has an important influence on methane adsorption and desorption behaviors^5^. The complexity of pore structure, morphology, pore size distribution, and connectivity in coal determine the storage and transportation of methane, controlling the storage pattern, diffusion path, and gas distribution of gas molecules in the complex pore network structure^6,7^. IUPAC pore classification method classifies pore structures with pore range larger than 50 nm as macropores, from 2 to 50 nm as mesopores, and less than 2 nm as micropores, as Fig. 1 shown. Methane adsorbed in coal exists in three states, namely, adsorbed, free, and dissolved states^8^, adsorption–desorption is a reversible physical process, and isothermal adsorption of methane is consistent with the Langmuir equation. The adsorption capacity of methane is closely related to the degree of metamorphism^9^, pore structure^10^ and reservoir pressure^11^.

Existing results have studied more on pore structure and adsorption–desorption characteristics of coal, which is still a hot spot of research at home and abroad. In this paper, we propose a new methane adsorption model from a microscopic point of view by taking the effect of pore structure evolution on methane adsorption as the research object, and explain the rule of solid–liquid–gas conversion of methane controlled by pore structure evolution in coals, to provide theoretical support for the formulation of reasonable and effective methane exploitation and utilization in coal and methane management programs.

## Experiment preparation

### Experimental sample preparation

In this study, the Faer Coal Mine (FR), Qinglong Coal Mine (QL), and Wenjiaba Coal Mine (WJ), which were significantly affected by geological structure were collected, and the sample information is shown in Table 1.

**Table 1:** Basic information about coal samples.

Sample	Coal scale	The coalfield is located	Coal formations
FR	Anthracite	Liupanshui coalfield	Permian Longtan Formation
QL	Anthracite	Qianbei coalfield	Permian Longtan Formation
WJ	Anthracite	Zhina coalfield	Permian Longtan Formation

**Figure 2:**
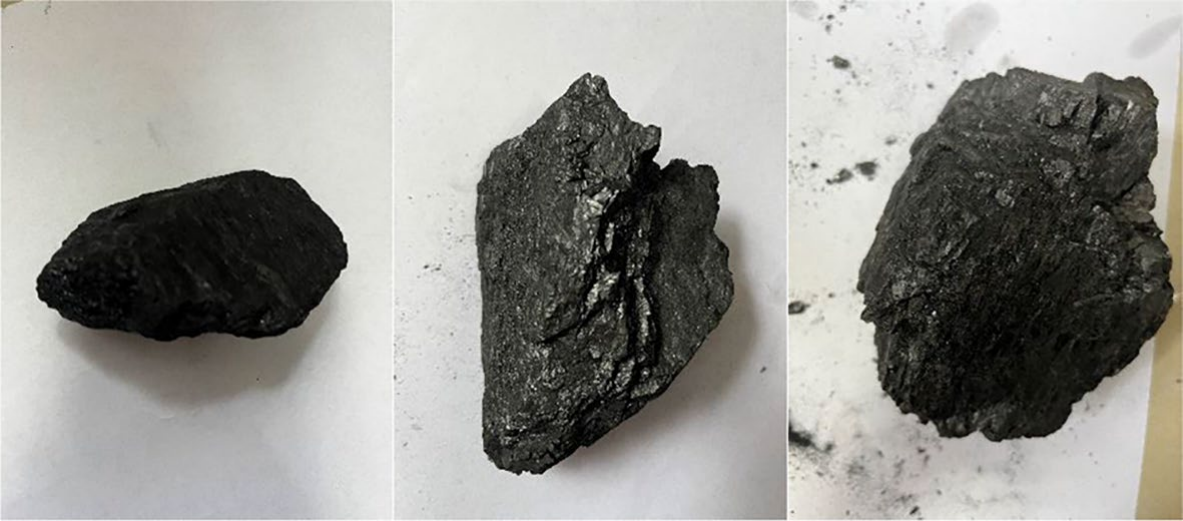
Macroscopic characteristics.

The macroscopic characteristics of the sample are shown in Fig. 2.

Elemental analysis of coal usually refers to the determination of organic matter element content in coal. In this paper, the elemental analysis of coal samples is based on the national standard GB/T31391-2015 (method of elemental analysis of coal), and the German Elementar Vario EL cube analyzer is used to determine the elemental information of coal samples, as shown in Table 2.

**Table 2:** Elemental analysis.

Samples	N (%)	C (%)	H (%)	S (%)
FR	1.169	68.037	2.324	0.000
QL	1.024	78.779	2.592	1.736
WJ	0.998	75.368	2.620	1.178

According to the requirements of GB/T30732-2014 (Coal industrial analysis method instrumental method), the test should include four parts: moisture, ash, volatile matter, and fixed carbon, and the test results are shown in Table 3.

**Table 3:** Industrial analysis.

Samples	M_ad_ (%)	A_ad_ (%)	V_ad_ (%)	FC_ad_ (%)
FR	1.08	12.35	18.49	68.08
QL	2.44	15.34	8.77	73.45
WJ	3.50	13.99	8.00	74.51

### Experimental test

**Figure 3:**
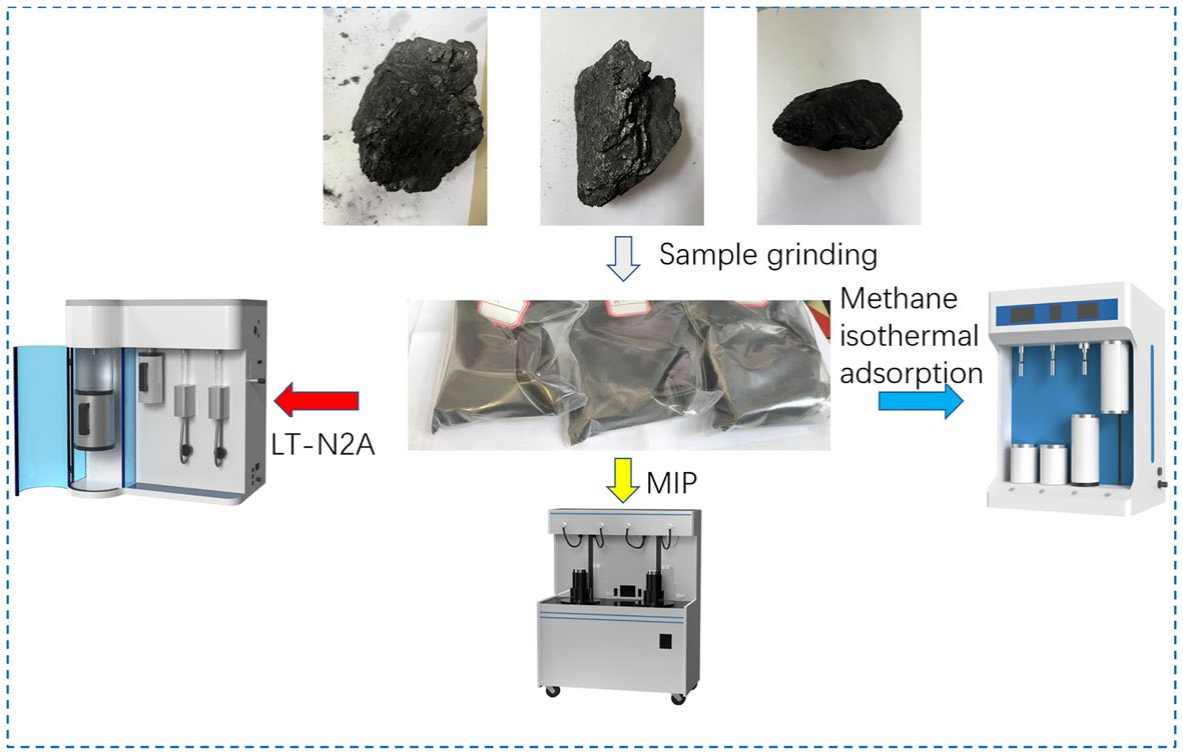
Test flow chart.

The experimental test flow is shown in Fig. 3.

#### LT-N_2_A experiments

For LT-N_2_A experiments, 0.25–0.3 mm coal samples were selected to avoid analyzing microscopic pore characteristics with coal samples of different sizes. Before the experiment started, the coal samples were placed in a drying oven and dried at 373 K for 6 h to prevent excessive moisture and impurities. Then 2 g of coal sample was weighed into the sample tube and mounted on the degassing unit of the analyzer. The sample tube was then mounted on the analyzer station for the LT-N_2_A experiment to determine the adsorption/desorption isotherms of the coal samples. The LT-N_2_A method was used to test the nanoscale pore size of coal in the range of 1.5–100 nm.

#### Mercury intrusion porosimetry (MIP) experiments

Test pressures range from 0.03 to 220 MPa and pore sizes range from 0.005 to 350 μm, the mercury intrusion pressure and null radius should be by the Washburn formula^14,15^:

1 $$\begin{array}{*{20}c} {r = - \frac{2\gamma cos\theta }{P}} \\ \end{array}$$

where, *r* is the pore radius; *γ* is the surface tension of mercury, 4.83 × 10^–3^ N/m; *θ* is the contact angle between mercury and the sample surface, 130°; and *P* is the pressure of mercury.

#### Methane isothermal adsorption experiment

Isothermal adsorption experiments were carried out using a PH1-1508-A high-pressure gas adsorption and constant pressure adsorption rate apparatus. This equipment carried out the methane adsorption and desorption experiments on three coal samples. The coal samples were crushed to less than 3 mm, and 50 g of each sample was taken for experimental measurement. After checking the airtightness of the equipment, the samples were measured to verify that the difference between the experimental data of each group and the a and b values measured by the parallel samples was not greater than 1, and the average value was taken as the measurement result; the temperature of the equipment was adjusted to 120 °C in the water bath, and the equipment was degassed under the vacuum condition for 180 min and then cooled down to 25 °C for the adsorption experiments after the degassing; the initial adsorption pressures of the instrument were set to 10 values, namely: 0.76 MPa, 1.85 MPa, 2.92 MPa, 3.94 MPa, 4.96 MPa, 5.97 MPa, 6.97 MPa, 7.94 MPa, 8.97 MPa, 9.86 MPa, and the adsorption equilibrium time was more than 12 h for each sample.

## Results and analysis

### Analysis of LT-N_2_A test results

The N_2_ adsorption and desorption isotherms were acquired in the relative pressure (P/P_0_) range between 0.01 and 0.99. Based on the adsorption branch of isotherms, the specific surface areas were analyzed by using the BET theory. Then, pore volumes and pore size distribution were analyzed by using the BJH theory^16^. The parameters of the coal samples measured in the test are shown in Table 4.

**Table 4:** The results of LT-N_2_A experimental.

Samples	BET surface area (m^2^/g)	Pore volume (mL/g)	Average pore radius (nm)
FR	6.252	0.0875	42.79
QL	4.607	0.0496	40.75
WJ	10.284	0.0302	45.10

**Figure 4:**
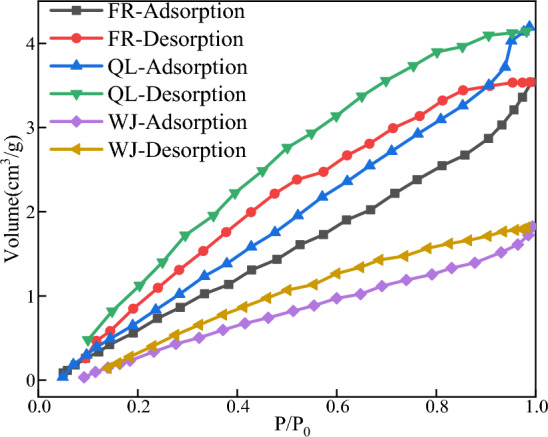
Nitrogen adsorption/desorption of isotherm curve.

The N_2_ adsorption and desorption isotherm curves^17^ are shown in Fig. 4.

As Fig. 4 shown, all samples show hysteresis loops with a wide range. In the initial stage of desorption, as the relative pressure decreases, the shape of the gas–liquid interface differs between coalescence and evaporation due to the presence of open pores, producing a return line.

The fractal theory was formalized by Mandelbrot^18^ and it is used to measure the complexity and structure of objects. The fractal dimension of a coal sample can be calculated from cryogenic liquid nitrogen experimental data. In this paper, The Frenkel–Halsey–Hill (FHH) model ^19,20^ is used to calculate the fractal dimension of coal samples. The calculation formula is as follows:

2 $$\begin{array}{*{20}c} {V \propto \left[ {\ln \left( {\frac{{P_{0} }}{P}} \right)} \right]^{D} .} \\ \end{array}$$

Derivation of (2) gives:

3 $$\begin{array}{*{20}c} {\ln V = c + D\ln \left[ {\ln \left( {\frac{{P_{0} }}{P}} \right)} \right],} \\ \end{array}$$

where, *V* (ml/g) is the volume of adsorption at balance pressure, *P*_*0*_ (MPa) is the saturation vapor pressure of gas adsorption, *P* (MPa) is balance pressure, *D* (dimensionless) is the fitting slope, and *c* is constant.

According to previous research results, the linear relationship was presented for fitting slope (*D*) and fractal dimension (*D*_*f*_), and fractal dimension was calculated by Eq. (4) as follows:

4 $$\begin{array}{*{20}c} {D_{f} = D + 3} \\ \end{array}$$

**Figure 5:**
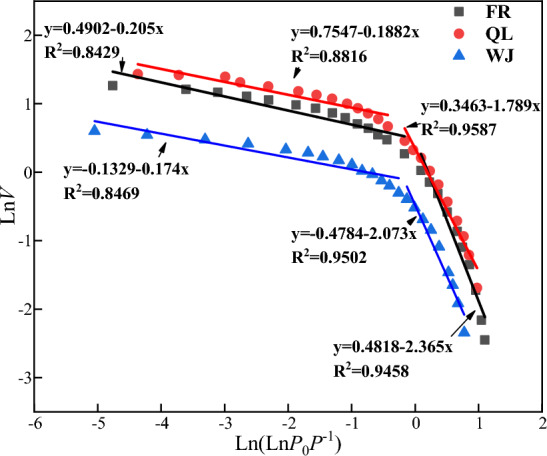
Fractal dimension fitting curve of LT-N_2_A experiment.

As shown in Fig. 5, the fractal dimension was calculated for different coal samples based on the fitting results of the fractal curves, and the correlation of the fractal dimension ranged from 0.8429 to 0.9587, shown in Table 5. Based on the fractal dimension calculations, it was found that the LT-N_2_A experiment was better at the Low-pressure stage, which could more accurately indicate the pore structure features.

**Table 5:** Fractal dimension calculation results.

Samples	Low pressure			High pressure		
	*D*	*D*_*f1*_	R^2^	*D*	*D*_*f2*_	R^2^
FR	− 0.188	2.812	0.8816	− 2.365	0.635	0.9458
QL	− 0.205	2.795	0.8429	− 1.789	1.211	0.9587
WJ	− 0.174	2.826	0.8469	− 2.073	0.927	0.9502

### Analysis of MIP test results

**Figure 6:**
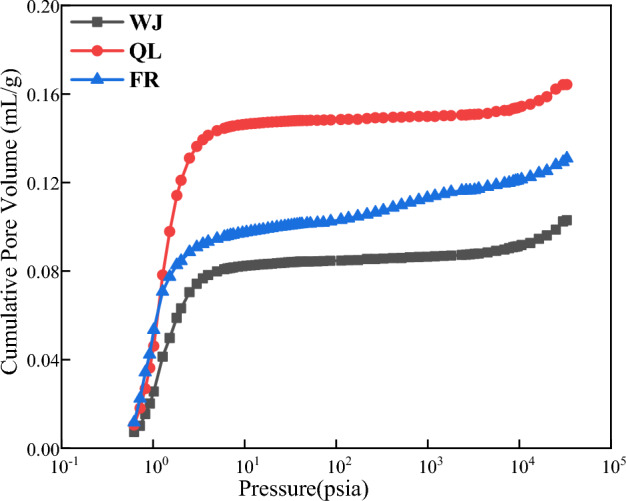
Mercury intrusion curves for samples.

The MIP curve can effectively and intuitively respond to the development status and connectivity of the internal pores of coal samples, and the mercury intrusion curve of the samples is shown in Fig. 6. From the beginning of applying pressure, the mercury inlet of the coal samples showed an increasing trend, indicating that the large pores, medium pores, transition pores, and micropores all contribute to the pore volume. When the mercury feed pressure is less than 10 psi, the mercury feed tends to increase rapidly, and at this stage, the mercury mainly invades some visible pores as well as fissures; when the mercury feed pressure is in the range of 10 ~ 4000 psia, and the invaded pore sizes are in the range from 300 to 46,000 nm, the mercury feed in all the samples maintains to increase, but the increase shows a slowing down tendency.

Based on the MIP data, the specific surface area and pore volume of various types of pores of the coal samples can be classified, as shown in Tables 6 and 7. The contribution of micropores and transition pores to the total pore specific surface area of the coal samples is over 96%, which is the main contributor to the specific surface area of the coal, and this result is consistent with the results of the specific surface area contribution obtained from the LT-N_2_A experiments.

**Table 6:** Pore surface area of coal samples.

Samples	Pore surface area/(m^2^/g)				
	Micropore	Transition pore	Mesopore	Macropore	Total
FR	10.801	6.651	0.554	0.079	18.085
QL	15.660	5.626	0.250	0.091	21.627
WJ	11.633	4.474	0.324	0.104	16.535

**Table 7:** The pore volume of coal samples.

Samples	Pore volume/(mL/g)				
	Micropore	Transition pore	Mesopore	Macropore	Total
FR	0.574	2.061	1.439	1.379	5.453
QL	0.860	3.086	2.224	2.200	8.370
WJ	0.400	1.347	0.948	0.929	3.624

From Table 7, it can be seen that the transition pore volume in all samples has the largest contribution to the total pore volume, reaching more than 36%.

### Joint pore analysis

To better study the pore and fracture characteristics, liquid nitrogen adsorption and high-pressure mercuric intrusion method can be used to study the pore distribution characteristics in the whole pore size range. The principle of joint pores is^21,22^:

1. LT-N_2_A method to test the microporous parameters, and MIP method to test the mesopore and macroporous parameters;
2. the location of the joint pore in the range of the two test methods, the joint pore location is located in the small pore section, and the same pore size near the pore specific surface area, pore volume increment difference is minimized.

**Figure 7:**
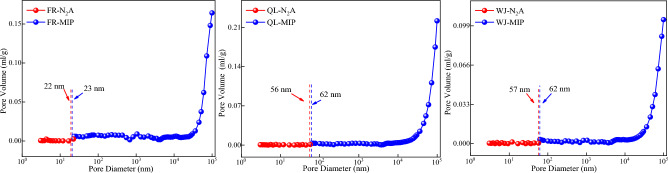
Pore volume distribution.

**Figure 8:**
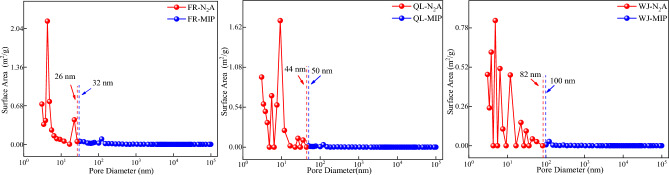
Specific surface area distribution.

According to the above principles, the experimental coal samples' pore volume distribution (Fig. 7) and specific surface area distribution (Fig. 8) in the full pore diameter range were obtained.

As can be seen from Fig. 7, the pore volume of the three groups of coal samples is 22–62 nm, and the proportion of pore volume in each stage of the coal samples is still dominated by the medium and large pores. The proportion of the micro-small pores does not change much, which indicates that the excessive pores play a dominant role in the pore volume of the coal samples. The results are close to those of the experimental results of the mercuric pressure method. Therefore, compared with liquid nitrogen adsorption, the mercuric pressure method is more suitable for testing the medium pores and the large pores.

As can be seen from Fig. 8, the pore specific surface area of the three groups of coal samples is 26–100 nm. The pore specific surface area of the coal samples is dominated by micropores in each stage. The percentage of medium and large pores does not change much, which indicates that micropores play a dominant role in the pore specific surface area of the coal samples, the result is similar to the results of liquid nitrogen adsorption experiments, so compared with the pressed mercury method, the liquid nitrogen adsorption method is more suitable for the testing of the pore specific surface area of the micro and small pores. Therefore, the LT-N_2_A method is more suitable for testing the pore specific surface area of micropores than the MIP method.

### Analysis of isothermal adsorption results

**Figure 9:**
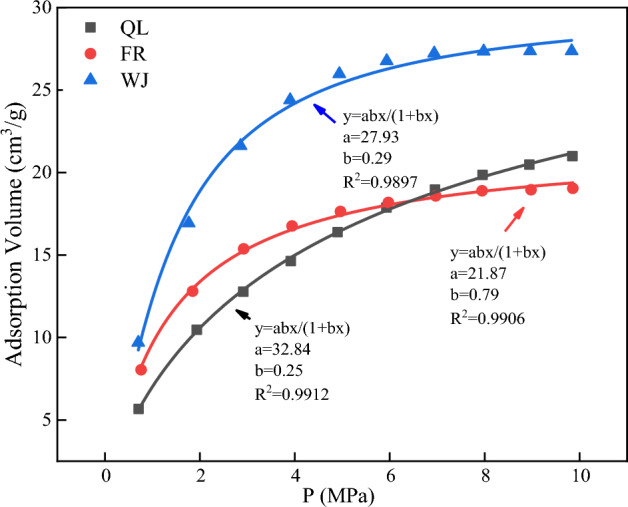
Isothermal adsorption curve.

The adsorption experimental temperature was 298.15 K, carried out at 10 pressures, and the adsorption equilibrium time of each sample was more than 12 h. The isothermal adsorption curves of the experimental coal samples are shown in Fig. 9, and the values of *a* and *b* and the correlation are shown in Table 8.

**Table 8:** Adsorption constant.

Samples	*a* (cm^3^/g)	*b* (MPa)	R^2^
QL	32.84	0.25	0.9912
FR	21.87	0.79	0.9906
WJ	27.93	0.29	0.9897

Figure 9 shows the adsorption capacity of coal samples increased with the increase of adsorption pressure, and gradually tended to slow down from the beginning of the rapid increase in the trend, and ultimately reached equilibrium; the analysis of the overall point of view shows that the saturated adsorption capacity of FR sample is the lowest, and the highest of WJ sample, and combined with the Tables 6 and 7, it can be seen that the specific surface area of the micropore and the ratio of the occupied area play important roles for the adsorption of methane by the coal.

Value a reflects the change of saturation adsorption amount, and with the increase or decrease of specific surface area and the change of microporous ratio, value *a* also changes accordingly, value *b* reflects the sensitivity of methane adsorption to gas pressure. The correlation coefficients are all above 98%, indicating that the fitting results are reliable.

## Correction of the adsorption model

### Langmuir adsorption model

In 1916, Langmuir proposed the theory of monolayer adsorption based on the gas–solid interface^23^. The basic assumption is that the isotropic properties of the adsorbent surface are uniform, and only one molecule can be adsorbed at an adsorption site on the adsorbent surface, there is no interaction force between molecules, and the adsorption is dynamic equilibrium, based on this, let the pressure of the gas be *p*, and the percentage of surface area not adsorbed by the gas molecules be *θ*_0_. The speed of adsorption of gas molecules is proportional to the pressure, and also proportional to the surface area not adsorbed by gas molecules, then the adsorption speed is formula (5):

5 $$\begin{array}{*{20}c} {R_{a} = cp\theta_{0} ,} \\ \end{array}$$

where, *c* is the scale coefficient, no factor.

The speed of gas desorption is proportional to the percentage of surface area adsorbed by gas molecules, and also proportional to the proportion of molecules adsorbed by gas molecules with the energy required for desorption. Let the percentage of surface area adsorbed by gas molecules be $\theta$, the $\varepsilon_{{\text{a}}}$ be heat adsorption, the total number of molecules adsorbed is $N_{{\text{a}}}$, The number of molecules adsorbed in the total number of molecules is n, and the number of molecules with adsorption heat exceeding $\varepsilon_{{\text{a}}}$ is $N_{{\text{a}}}^{*}$, then there are:

6 $$\begin{array}{*{20}c} {N_{{\text{a}}}^{*} /N_{{\text{a}}} = fe^{{\varepsilon_{{\text{a}}} /kT}} ,} \\ \end{array}$$

where, *f* is scale coefficient, no factor; *k* is the Boltzmann constant, 1.38 × 10^–23^ J/*K*。

Then the desorption speed:

7 $$\begin{array}{*{20}c} {R_{d} = d\theta {\text{e}}^{{\varepsilon_{{\text{a}}} /kT}} ,} \\ \end{array}$$

where, *d* is the scale coefficient, no factor.

When the adsorption equilibrium is reached, the adsorption velocity should be equal to the desorption velocity, that is $R_{{\text{a}}} { = }R_{{\text{d}}}$, obtained:

8 $$\begin{array}{*{20}c} {cp\theta_{0} = d\theta e^{{\varepsilon_{a} /kT}} .} \\ \end{array}$$

The sum of the percentage of surface area θ not adsorbed by gas molecules and the percentage θ of surface area adsorbed by gas molecules shall be equal to 1.

9 $$\begin{array}{*{20}c} {\theta_{0} + \theta = 1.} \\ \end{array}$$

Substituting formula (9) into formula (8) is the single-layer adsorption formula;

10 $$\begin{array}{*{20}c} {\theta = \frac{bp}{{1 + bp}},} \\ \end{array}$$

where, *b* is the adsorption coefficient.

If *V* represents the amount of adsorbed gas on the unit solid surface, and a represents the amount of saturated adsorbed gas on the unit solid surface, it is *a* common form of the langmuir formula:

11 $$\begin{array}{*{20}c} {V = \frac{abp}{{1 + bp}}.} \\ \end{array}$$

Sips et al. established the Langmuir–Freundlich adsorption model^24,25^, which considered the interaction between adsorbed molecules.

12 $$\begin{array}{*{20}c} {V = \frac{{V_{{\text{L}}} \left( {bp} \right)^{n} }}{{1 + \left( {bp} \right)^{n} }},} \\ \end{array}$$

where, *V*_L_ is the Langmuir adsorption volume; n is the model parameter related to the non-uniformity or heterogeneity of the adsorbent, and the value range is 0 ~ 1.

According to the effect of pore size distribution on adsorption, formula (12) is modified to:

13 $$\begin{array}{*{20}c} {V = \frac{{h_{1} V_{{\text{L}}} \left( {b_{1} p} \right)^{n} }}{{1 + \left( {b_{1} p} \right)^{n} }} + \frac{{h_{2} V_{{\text{L}}} \left( {b_{2} p} \right)^{n} }}{{1 + \left( {b_{2} p} \right)^{n} }},} \\ \end{array}$$

where, $h_{1} , h_{2}$ represent the proportion coefficient of Langmuir adsorption volume $V_{{\text{L}}}$ under different pore size distributions, the value range is 0 ~ 1, and the sum of the two is 1.

### Adsorption data fitting

The modified LF adsorption model was used to fit the isothermal data of structural coal methane adsorption^26^, including the isothermal adsorption experimental data of three coal samples at 25 °C (K = 298.15). In this study, to evaluate the effect of the modified L–F equation fitting, the coefficient of determination *R*^2^ was selected as the evaluation criterion.

**Figure 10:**
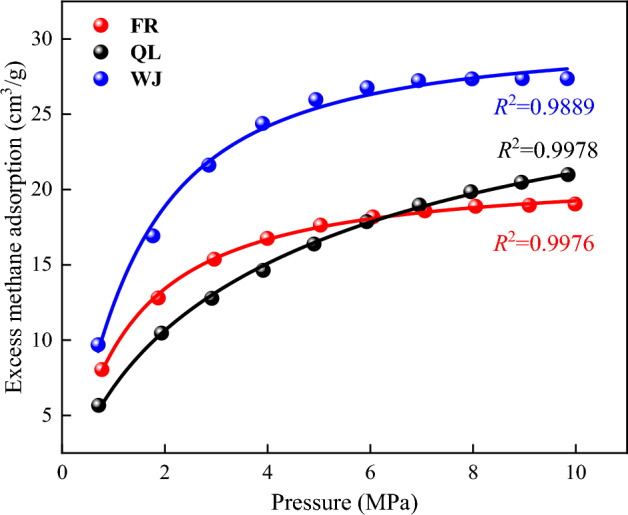
Fitting results of isothermal adsorption data of L–F equation after correction.

The isothermal adsorption data of three experimental coal samples were fitted by equation, and the fitting results were shown in Fig. 10, and the fitting parameters were shown in Table 9. The modified L–F equation is consistent with the isothermal adsorption lines fitted to the experimental coal samples in three groups and the measured data. It can also be seen that the fitting coefficient of the L–F equation after correction is greater than 0.9889, indicating that the modified L–F equation has a good fitting effect on the methane isothermal adsorption line of the three groups of experimental coal samples.

**Table 9:** Modified L–F equation parameters for fitting isothermal adsorption data of experimental coal samples.

Samples	Temperature/K	$V_{{\text{L}}}$	$h_{1}$	$h_{2}$	$b_{1}$	$b_{2}$	$n_{1}$	$n_{2}$
FR	298.15	21.6109	0.7941	0.2059	0.5882	0.5103	0.7988	2.7164
QL		29.4343	0.0471	0.9529	0.1904	0.2768	6.7243	0.8429
WJ		32.0492	0.0247	0.9753	0.5036	0.8359	2.9109	0.6052

Table 9 shows the 6 parameters ($h_{1} ,{ }h_{2} ,{ }b_{1} ,{ }b_{2} ,{ }n_{1}$, $n_{2}$) of the modified L–F fitting, from which it can be seen that at the same temperature, the Langmuir adsorption volume of the WJ coal sample is the largest, followed by QL, and the FR is the smallest. The modified model reflects the presence of adsorbed methane molecules with different adsorbed phases in different pore structures.

## Molecular simulation

### The molecular configuration of the structural coal

Combined with previous studies, it can be seen that coal mainly controls methane desorption through pore structure^27–29^, so it is advisable to use the representative W.Fuchs model to form coal slits and further simulation^30–32^. The model considered the morphology of C, O, H, S, and N atoms and the size of aromatic nuclei in coal, among which the molecular formula of the structural coal was C_134_H_101_NO_9_S (C) accounting for 84.99%, H accounting for 5.04%, O accounting for 7.56%, N accounting for 0.73 and S accounting for 1.68%^33–36^.

### Effect of pore evolution on methane adsorption

**Figure 11:**
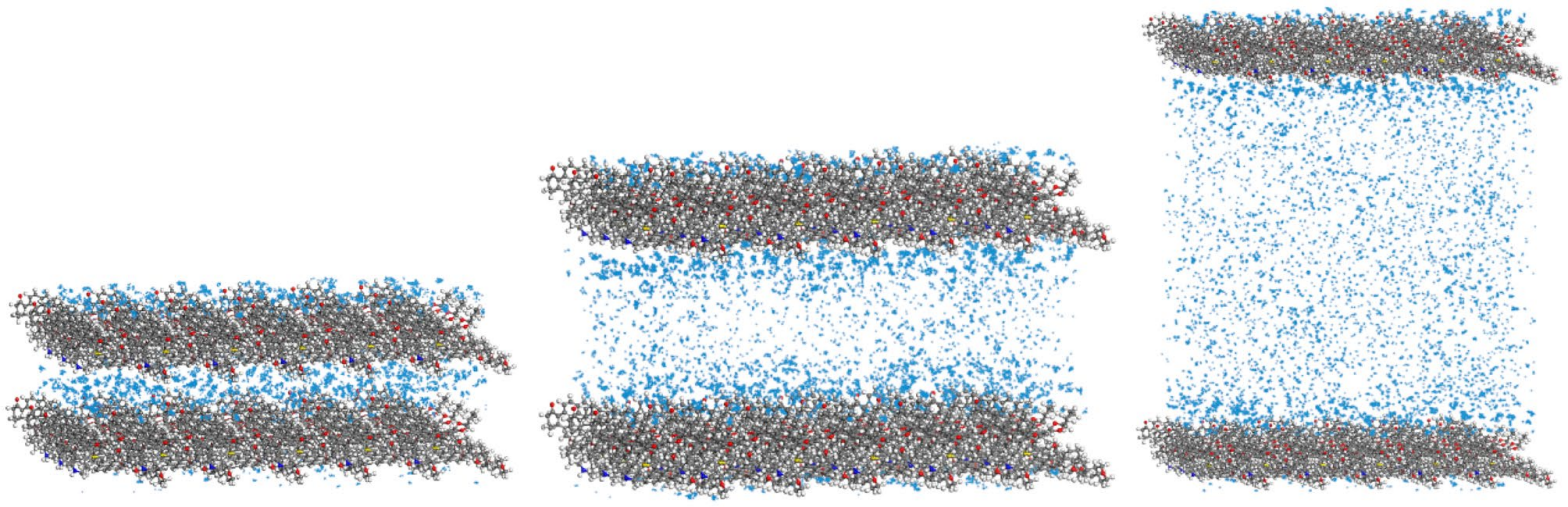
Density distribution of methane under different pore size structures.

At an initial temperature of 298 K, the methane fugacity was set to 5 MPa and filled into the 2 nm, 4 nm, and 10 nm slit holes, and the distribution of methane at different fugacity and pore size was tested. Since there are many control experiments, only part of the simulation results are shown, and the simulation results are shown in Fig. 11.

Analysis of the above figure shows that with the increase of fugacity, methane molecules are preferentially adsorbed on the surface of the slit pores, and are centrally symmetrically distributed in the pores, with larger methane densities on both sides of the slit pores and smaller methane densities in the slit pores. Because the methane molecules on the surface of the pore wall of the slit are most strongly adsorbed, a dense adsorption layer is formed, while the methane density is lower near the middle of the pores far from the wall. When the pore size of the slit is 2 nm, the methane molecules are strongly adsorbed by the pore wall, and the space in the 2 nm slit pore is limited, and the diffusion capacity of methane molecules is limited, so it is mainly adsorbed. Observing the 4 nm slit hole, it can be seen that because the methane molecule located in the center of the pore is farthest from the wall of the slit, it is less restrained, and free state molecules appear, but the overall methane molecule is in the adsorbed state. Observation of the 10 nm slit pores shows that with the expansion of pore size, the number of free molecules increases, and there are more free molecules in the pore size than in the adsorbed state. In summary, it can be seen that the methane molecules in the slit pores also change accordingly with the change in the pore structure.

**Figure 12:**
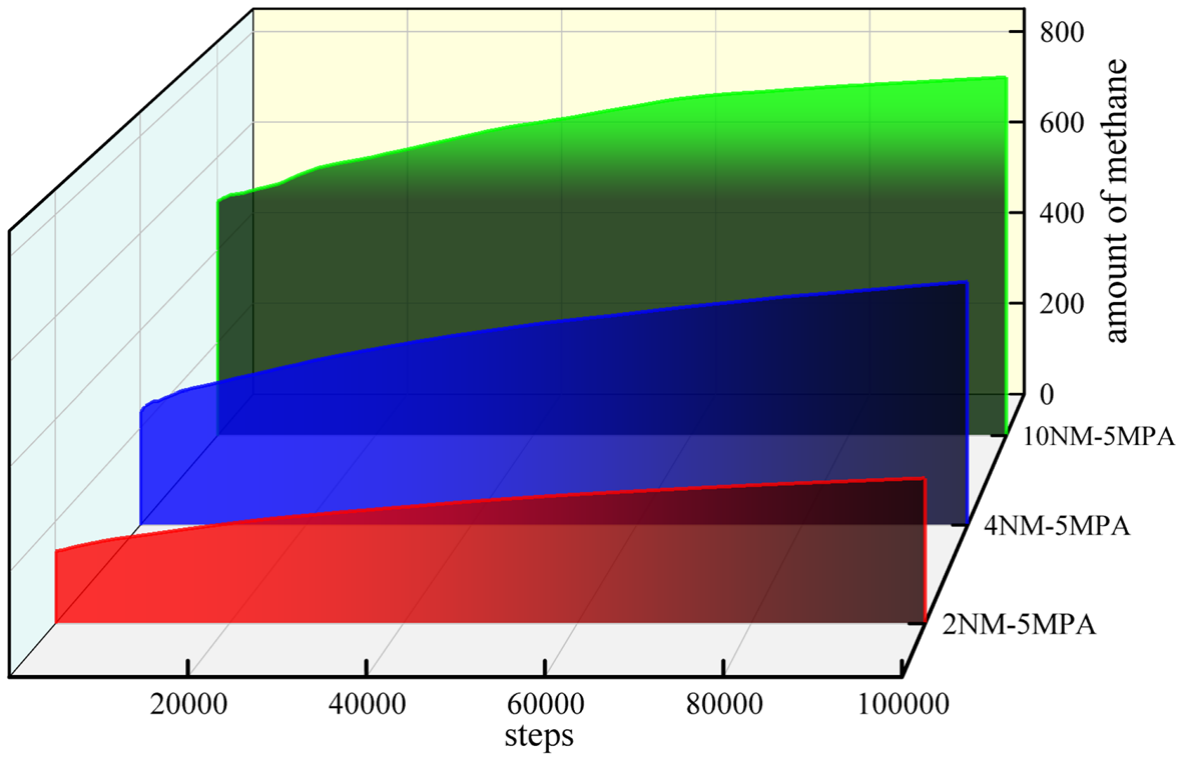
Methane adsorption in different slit structures.

At an initial temperature of 298 K, the methane density distribution at different pore size structures was tested. As shown in Fig. 12.

The number of methane molecules adsorbed by unit cells fluctuates not much with the increase of fugacity, which is due to the small size of the 2 nm slit pores, the limited diffusion of methane, and the adsorption state of most methane molecules closer to the wall of the slit pore. The comparison shows that the 4 nm and 10 nm slits pressurize methane at the same time, and the adsorption of methane molecules in the unit cell increases, and under the same conditions, the amount of methane adsorbed in the 4 nm and 10 nm slits is greater than that of the 2 nm slits. In terms of total adsorption per unit cell, the adsorption capacity increased from 285.2 to 772.8 when the slit pores were in the range of 2–10 nm at 5 MPa. It was shown that the larger the pore size, the greater the methane adsorption capacity, and with the increase of fugacity, the greater the influence of pore size change on the adsorption capacity, and the pore size was proportional to the methane adsorption capacity.

**Figure 13:**
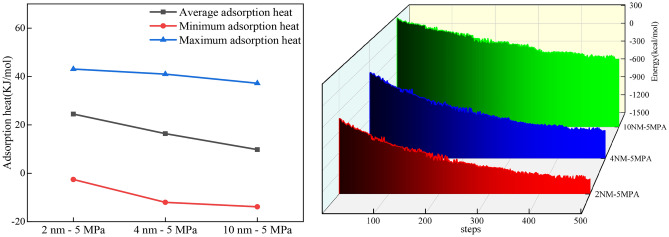
Methane molecule adsorption heat and energy distribution under different pore sizes.

Studies have shown that the methane adsorption process is an exothermic reaction, and exothermic heat will cause a decrease in the energy of the system. At an initial temperature of 298 K, the potential energy changes of slit holes under different conditions were tested separately shown in Fig. 13.

With the continuous compression of the total potential energy of methane molecules in the continuous compression slit, the potential energy of the 2 nm slit hole was 1275.9 kcal/mol, the 4 nm slit hole was 1064.9 kcal/mol, and the 10 nm slit hole was 386.9 kcal/mol. Combined with the methane adsorption heat under different pore sizes and fugacities in Fig. 13, it can be seen that the methane molecule has the largest adsorption heat in the 2 nm slit hole under the same fugacity, which is 1.49 times that of the 4 nm slit hole on average, and the 4 nm slit hole is followed by 1.67 times that of the 10 nm slit hole. The heat of adsorption can accurately express the physical or chemical nature of the adsorption phenomenon, the activity of the adsorbent, and the strength of the adsorption capacity. The heat generated by the adsorption process is the size of the adsorption heat, which can measure the degree of adsorption strength, and the greater the adsorption heat, the stronger the adsorption. Therefore, it can be seen that the 2 nm slit pore has maximum adsorption capacity, followed by 4 nm slit pores, and the 10 nm slit pore has minimal adsorption capacity.

**Figure 14:**
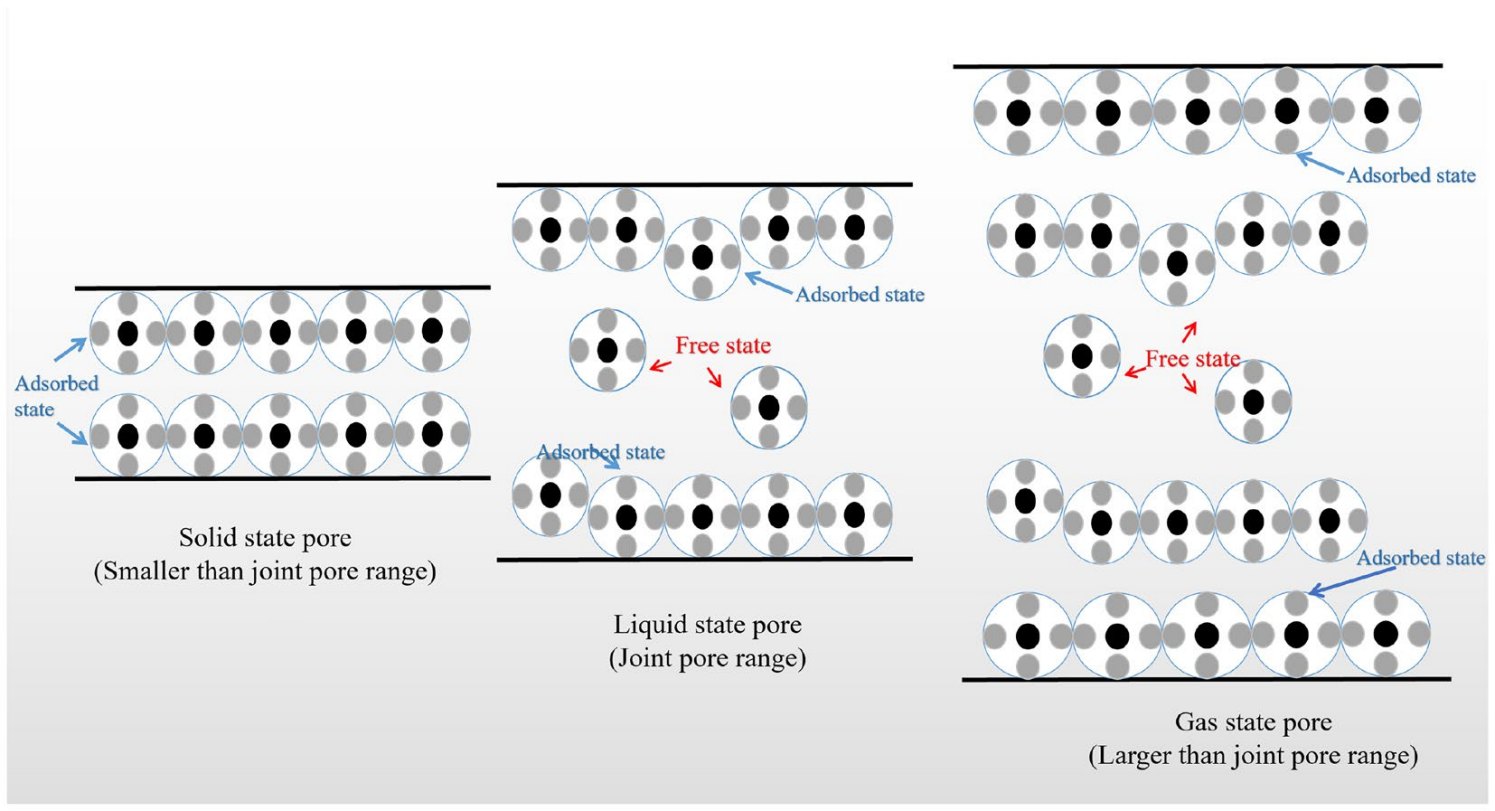
Schematic diagram of methane solid–liquid–gas conversion in pore structure.

Combined with the adsorption model and simulation results, a single adsorption mode can not reasonably explain the adsorption state of coal, starting from the pore characteristics of coal, there is a critical pore size in the pores of coal. Pore size smaller than the range of joint pores that can be filled by methane molecules are defined as solid state pores; pore size within the range of joint pores with both free and adsorbed gas are defined as liquid state pores; and pore size larger than the range of joint pores with freer than adsorbed gas are defined as gas state pores, as shown in Fig. 14.

## Conclusion

1. MIP and LT-N_2_A experiments showed a high percentage of medium and large pores in the pore volume and specific surface area measured by the high-pressure mercury pressure method and a high percentage of micropores and small pores in the pore volume and specific surface area measured by liquid nitrogen adsorption method. According to the principle of the joint pore, the pore volume joint pores were 22–62 nm, and the pore specific surface area joint pores were 26–100 nm, respectively.
2. The L–F model was modified according to the pore size distribution, and the coal pore binding position was obtained as the critical pore size for the three-phase methane solid–liquid–gas transition. The modified L–F model can fit the three sets of isothermal adsorption data well which fit a degree higher than 0.9889.
3. The scale factor *h*_1_ is positively correlated with the specific surface area of medium and large pores after the cascade, and *h*_2_ is positively correlated with the specific surface area of small pores after the cascade so that methane molecules under different pore sizes undergo solid–liquid–gas transition at cascade positions.
4. According to the molecular simulation results, solid–liquid–gas three-phase conversion of methane molecules occurred at different pore sizes. Under the same injection fugacity and 5 MPa pressure condition, according to the number of methane molecules, the heat of adsorption, and energy, it can be seen that the 10 nm slit pore adsorbs the largest number of methane with the weakest adsorption capacity, and the 2 nm slit pore has the smallest adsorption amount but the strongest adsorption capacity.
5. Pore size smaller than the range of joint pores that can be filled by methane molecules are defined as solid state pores; pore size within the range of joint pores with both free and adsorbed gas are defined as liquid state pores; and pore size larger than the range of joint pores with freer than adsorbed gas are defined as gas state pores.

## Data Availability

All data generated or analysed during this study are included in this published article.
